# Non-Targeted Metabolomics Analysis of *γ*–Aminobutyric Acid Enrichment in Germinated Maize Induced by Pulsed Light

**DOI:** 10.3390/foods13172675

**Published:** 2024-08-24

**Authors:** Liangchen Zhang, Xiaojing Liu, Liwei Xu, Mengxi Xie, Miao Yu

**Affiliations:** 1 Institute of Food and Processing, Liaoning Academy of Agricultural Sciences, Shenyang 110161, China; 2 Center for Disease Control and Prevention of Liaoning Province, Shenyang 110172, China

**Keywords:** non-targeted metabolomics, *γ*–aminobutyric acid, maize, pulsed light, germination, phytohormones

## Abstract

Pulsed light is an emerging technique in plant physiology recognized for its ability to enhance germination and accumulate *γ*–aminobutyric acid in maize. Pulsed light involves exposing plants to brief, high-intensity bursts of light, which can enhance photosynthesis, improve growth, and increase resistance to environmental stresses. Despite its promising potential, the specific metabolic changes leading to *γ*–aminobutyric acid enrichment in maize induced by pulsed light are not fully understood. This study addresses this gap by quantifying key nutrients and *γ*–aminobutyric acid-related compounds during maize germination and investigating the underlying mechanisms using non-targeted metabolomics. Our findings indicate that pulsed light significantly promotes maize germination and accelerates the hydrolysis of proteins, sugars, and lipids. This acceleration is likely due to the activation of enzymes involved in these metabolic pathways. Additionally, pulsed light markedly increases the content of glutamic acid and the activity of glutamate decarboxylase, which are crucial for *γ*–aminobutyric acid synthesis. Moreover, pulsed light significantly reduces the activity of *γ*–aminobutyric transaminase, thereby inhibiting *γ*–aminobutyric acid decomposition and resulting in a substantial increase in *γ*–aminobutyric acid content, with a 27.20% increase observed in germinated maize following pulsed light treatment. Metabolomic analysis further revealed enrichment of metabolic pathways associated with *γ*–aminobutyric acid, including amino acid metabolism, carbohydrate metabolism, plant hormone signal transduction, energy metabolism, pyrimidine metabolism, and ABC transporters. In conclusion, pulsed light is a robust and efficient method for producing sprouted maize with a high *γ*–aminobutyric acid content. This technique provides a novel approach for developing sprouted cereal foods with enhanced nutritional profiles, leveraging the physiological benefits of *γ*–aminobutyric acid, which include stress alleviation and potential health benefits for humans.

## 1. Introduction

Maize (*Zea mays* L.) is a significant food crop renowned for its rich composition of carbohydrates, proteins, fats, carotenoids, and various nutrients [1]. It is the most productive crop worldwide now, and it is widely used as food, feed, and raw materials for various industrial products. However, the amino acid composition of maize seeds is considered suboptimal, resulting in a mere 6% utilization rate in the food industry [2]. Therefore, it is important to leverage its nutritional quality in other ways. For example, the germination of maize seeds has been shown to enhance their nutritional profile by partially degrading proteins and carbohydrates, increasing soluble components, and improving the content of vitamins and soluble dietary fibers. This process also reduces product viscosity, enhances rehydration, and imparts a unique sweet and aromatic flavor, thereby enhancing consumer appeal. Germination can significantly increase the *γ*–aminobutyric acid (GABA) content in maize, mainly because during the germination process, the glutamic acid decarboxylase (GAD) of maize seeds is activated, causing endogenous glutamic acid (Glu) decarboxylation to produce GABA. The protein hydrolysis of maize seeds can also produce GABA. The metabolism and synthesis of GABA in plants are closely related to the action of enzymes such as GAD, *γ*–aminobutyric transaminase (GABA–T), and succinic semialdehyde dehydrogenase. Studies have found that external physical field treatments such as ultrasound, microwave, magnetic field, and light can enhance the activity of glutamate decarboxylase in plant seeds, thereby enriching GABA in grains or shortening the enrichment time of GABA [3].

GABA, a non-protein amino acid prevalent in both plants and animals, has many physiological effects on the human body [4]. The blood pressure-lowering function is the most important physiological function of GABA. When combined with postsynaptic GABA_A_ receptors that dilate blood vessels and GABA_B_ receptors that inhibit sympathetic nerve endings, GABA can effectively promote blood vessel dilation, thereby achieving the goal of lowering blood pressure [5]. GABA has always been believed to improve sleep, and some drugs can increase its content in the brain by inhibiting the breakdown of GABA in the human body, which can to some extent increase the body’s sleep time [6]. GABA also has other functions, such as promoting the secretion of growth hormone, regulating insulin secretion to prevent diabetes, enhancing memory, reducing blood lipid levels, preventing obesity, inhibiting cancer cell proliferation, promoting alcohol metabolism, and alleviating depression [7]. Typically, plants contain low levels of GABA under normal growth conditions. However, studies have shown that the GABA content in rice [8], soybean [9], and wheat [10] can increase several-fold when these plants are germinated under adverse conditions such as low oxygen, low temperature, salt stress, heat stress, mechanical damage, or light stress.

Pulsed light (PL) is a novel non-thermal treatment technology that mimics the wavelength spectrum of sunlight, covering ultraviolet, visible, and infrared regions [11]. Characterized by its high energy and short duration, PL is utilized for the rapid inactivation of pathogenic and spoilage microorganisms in food [12]. Recent studies have demonstrated that when applied to plant seeds, PL can enhance enzyme activity, promote cell growth, and increase GABA content [13].

Metabolomics, which analyzes changes in metabolites in organisms, has been extensively employed to monitor seeds under various stress conditions such as heat, cold, drought, and salt [14,15,16]. However, the metabolomic mechanism of PL treatment on GABA enrichment during maize seed germination has not been reported. This study investigated changes in the contents of basal nutrients such as free amino acids (FAAs), GABA, and Glu, as well as the activities of GAD and GABA–T in germinated maize seeds subjected to PL treatment across different germination periods. Using a non-targeted metabolomics approach, this study identified metabolites that differed between PL-treated and untreated germinated maize, analyzed the differential metabolites and metabolic pathways of germinated maize before and after PL treatment, and analyzed the correlation between various metabolic pathways and GABA synthesis. The findings elucidate how PL treatment influences *γ*–aminobutyric acid metabolism in germinated maize seeds, offering new insights for the development of functional maize products and expanding the application of pulsed light technology to enhance the nutritional value of germinated grains.

## 2. Materials and Methods

### 2.1. Materials and Reagents

Maize (Liaodan 575, developed by the Corn Research Institute of Liaoning Academy of Agricultural Sciences and widely planted in Liaoning Province, China), provided by the Maize Research Institute of Liaoning Academy of Agricultural Sciences, was harvested in October 2023, threshed and saved in airtight containers, and stored at 4 °C. NaClO, potassium phosphate buffer, and *α*–ketoglutarate were purchased from Solarbio (Beijing Solarbio Science & Technology Co., Ltd., Beijing, China); GABA, Glu, and nicotinamide adenine dinucleotide were purchased from Sigma–Aldrich (Sigma–Aldrich, St. Louis, MO, USA); L–2–chlorophenylalanine was purchased from Adamas–beta (Adamas–beta, Shanghai, China); methanol, acetonitrile, and formic acid were HPLC-grade and purchased from Fisher Chemical (Thermo Fisher Scientific, Waltham, MA, USA).

### 2.2. Surface Sterilization of Seeds

The soaking treatment was carried out as outlined by Hiran et al. [17], with modifications. A total of 800 g of maize seeds were weighed and sterilized by soaking in a 1% by volume NaClO solution for 30 min, rinsed with deionized water to restore a neutral pH, Subsequently, the seeds were immersed in distilled water at a ratio of 1:6 (seed:water) for 6 h at 30 °C in an incubator.

### 2.3. Pulse Light Treatment

After sterilization, 400 g of maize seeds were spread evenly on a quartz plate (30 cm × 30 cm) and treated with pulsed light (PL) using a surface sterilization machine (ZWS–Y1–D2, Zhongwu, Ningbo, Zhejiang, China). The PL treatment parameters were set at 400 pulses, with a light intensity of 0.50 J/cm² per pulse and a treatment distance of 7.0 cm from the light source. The maize seeds germinated after the PL treatment, and the duration of the PL treatment was excluded from the total germination time. Maize seeds that have not been treated with PL as a control (CK) for subsequent experiments.

### 2.4. Germination

PL and CK groups of maize seeds were separately placed in Petri dishes and kept at a constant temperature of 30 °C and 90% relative humidity for 72 h. During the germination process, 100 g of samples were collected from the PL group and the CK group at 0, 24, 48, and 72 h. Each 100 g sample was divided into two 50 g portions. One portion was rinsed three times with distilled water, drained, and stored in sterile bags at −80 °C for metabolomics analysis. The other portion was crushed, freeze-dried to less than 10% moisture content, and stored at 4 °C for the determination of FAAs and GABA contents, as well as GAD and GABA–T activities.

### 2.5. Macromolecule and γ–Aminobutyric Acid Quantification

Reducing sugar content was determined using the 3, 5–dinitrosalicylic acid method [18]. Soluble protein content was measured using the Bradford method [19], and fat content was determined by the Soxhlet extraction method [20]. To quantify GABA and FAAs in germinated maize, the test method adapted the method described by Chen et al. [21] with modifications. A 1.0 g sample of germinated maize powder was mixed with 5 mL of deionized water in a test tube and extracted at 40 °C and 45 kHz for 30 min using a Sonoswiss ultrasonic extractor (PRESET–SW–12H; Sonoswiss, Switzerland). The extract was then centrifuged at 8000× *g* for 5 min, and the supernatant was collected. This process was repeated once, and the supernatants from the two extractions were combined. Next, 1.5 mL of the combined supernatant was mixed with 3.5 mL of 100% ethanol and left at 4 °C for 12 h, followed by another centrifugation at 8000× *g* for 10 min. The supernatant was then evaporated using a dry nitrogen blower (DN–12A; Bilang, Shanghai, China). The residue was reconstituted in 1 mL of 0.02 M HCl, centrifuged at 8000× *g* for 10 min, and the final supernatant was filtered through a 0.45 μm filter (Shanghai Jinlan Instrument Manufacturing Co. Ltd., Shanghai, China). The GABA and free amino acid contents were determined using an automated amino acid analyzer (L–8900, Hitachi, Tokyo, Japan).

### 2.6. Determination of Glutamate Decarboxylase Activity

GAD activity was determined according to the method described by Khwanchai et al. [22], with some modifications. Maize flour was suspended in 0.05 M potassium phosphate buffer (pH 5.8) and centrifuged at 1000× *g* for 20 min. The obtained supernatant is the GAD extract. Next, 0.2 mL of GAD extract and 0.1 mL of 1% Glu are mixed and incubated at 40 °C for 2 h. The enzyme reaction was terminated by heating at 90 °C for 10 min and the GABA content was determined as described above. One unit (U) of GAD activity was defined as the amount of enzyme required to produce 1 μmol of GABA per minute at 40 °C.

### 2.7. Determination of γ–Aminobutyric Transaminase Activity

The activity of GABA–T was determined following a modified protocol from Huang et al. [23], with some modifications. An array of nicotinamide adenine dinucleotide standard solutions were prepared by dissolving the appropriate amounts of nicotinamide adenine dinucleotide disodium salt (NADH) in deionized water to achieve concentrations of 0, 4, 8, 16, 32, and 64 μg/mL. For the enzyme assay, aliquots of the standard solution were mixed with potassium phosphate buffer (pH 8.75, containing 0.001 M nicotinamide adenine dinucleotide (NAD+)) and substrate (4.8 mM *α*–ketoglutarate) and 18 mM GABA at a ratio of 1:1, pH 8.75). The mixtures were incubated in a water bath at 30 °C for 30 min, and the absorbance was measured at 340 nm using an ultraviolet spectrophotometer (UV755B, Youke, Shanghai, China) to construct the standard curve.

To prepare the crude enzyme solution, 0.5 g of germinated maize powder was mixed with potassium phosphate buffer (10 mM, pH 6.8) and incubated in an ice bath for 10 min. The mixture was then filtered to obtain the crude enzyme solution. A portion of the crude enzyme solution was centrifuged, and the supernatant was used for the enzyme activity assay. The supernatant was mixed with potassium phosphate buffer and substrate, and the reaction was carried out in a water bath at 30 °C for 30 min. The absorbance was measured at 340 nm, and the NADH content was calculated from the standard curve. Enzyme activity was expressed as units (U), defined as the amount of enzyme required to produce 1 μmol of NADH per minute.

### 2.8. Metabolomic Sample Extraction

Metabolomics analyses were carried out on each group of germinated maize, with significant differences observed in chemical substances. The different treatments and controls were named as follows: maize seeds germinated under normal conditions for 24 h, 48 h, and 72 h were denoted as CK24, CK48, and CK72, respectively, and maize seeds germinated after PL treatment for 24 h, 48 h, and 72 h were denoted as PL24, PL48, and PL72, respectively.

Germinated maize was mixed in a centrifuge tube with a 4:1 methanol to water ratio (v:v) that contained 0.02 mg/mL of the internal standard (L–2–chlorophenylalanine). Samples were ground for 6 min (-10 °C, 50 Hz) with a tissue grinder (Wonbio–96c, Wonbio, Shanghai, China) and extracted by an ultrasonic apparatus (PRESET–SW–12H, Sonoswiss, Switzerland) at 5 °C, 40 kHz for 30 min. The extract was centrifuged at 13,000× *g* for 15 min at 4 °C, and the resulting supernatant was utilized for LC/MS analysis.

### 2.9. HPLC–MS/MS Analysis

Metabolite analysis was conducted with an ultra-high-performance liquid chromatography tandem Fourier transform mass spectrometer (UHPLC–Q Exactive HF–X, Thermo Fisher Scientific, Waltham, MA, USA) with an HSS T3 column (100 mm × 2.1 mm i.d. × 1.8 μm; Waters, Milford, CT, USA). The column temperature was maintained at 30 °C with a flow rate of 0.3 mL/min. Mobile phases consisted of A (water with 0.1% v/v formic acid) and B (acetonitrile). The injection volume was 2 μL, and the autosampler temperature was held at 4 °C. Mass spectrometry was performed in both positive and negative ion scanning modes, with a mass scanning range of 70–1050 m/z. The sheath gas flow rate was 50 psi, the auxiliary gas flow rate was 13 psi, and the auxiliary gas heating temperature was 425 °C. The capillary temperature was 425 °C, and the spray voltages were 3500 V in positive mode and −3500 V in negative mode. The normalized collision energy was 20–40–60 eV, and the resolution was 60,000 for the primary mass spectrum and 7500 for the secondary mass spectrum. Data were collected in a data-dependent acquisition (DDA) mode. Electrospray ionization (ESI) positive and negative ion modes were used for detection. The instrument was calibrated using a calibration solution, and analyses were performed in full scan mode (FM). Quality control (QC) samples, prepared by mixing extracts of all samples in equal volumes, were analyzed every 5–15 samples to ensure the reliability and stability of the results. The injection and detection methods for QC samples were consistent with those applied to normal samples.

### 2.10. Data Processing and Annotation

Results are presented as mean ± standard deviation (SD) (n = 6). Data processing and plotting were performed using SPSS 17.0 (SPSS Inc., Chicago, IL, USA) and Origin 2021 (OriginLab, Northampton, MA, USA). LC–MS/MS data were processed using Progenesis QI software 2.0 (Waters Corporation, Milford, CT, USA), which facilitated baseline filtering, peak identification, integration, retention time correction, and peak alignment. The data were filtered to remove missing values and variables with a relative standard deviation (RSD) > 30% in QC samples. The log10 transformation was applied to the data for subsequent analysis. Metabolite identification was conducted by matching MS and MS/MS information with the HMDB (http://www.hmdb.ca/), Metlin (https://metlin.scripps.edu/), and internal libraries. The preprocessed data matrix was analyzed using principal components analysis (PCA) and partial least squares discriminant analysis (OPLS–DA) in the ropls package in R (Version 1.6.2). Significantly different metabolites were identified based on variable importance in projection (VIP) scores (VIP > 1) and Student’s *t*-test *p*-values (*p* < 0.05). Pathway analysis was performed using the Kyoto Encyclopedia of Genes and Genomes (KEGG) 1.0.0 database (https://www.kegg.jp/kegg/pathway.html) for metabolic pathway annotation [24].

## 3. Results and Discussion

### 3.1. Effect of Pulsed Light Treatment on Morphological Characteristics and Basal Nutrients in Germinated Maize

**Figure 1:**
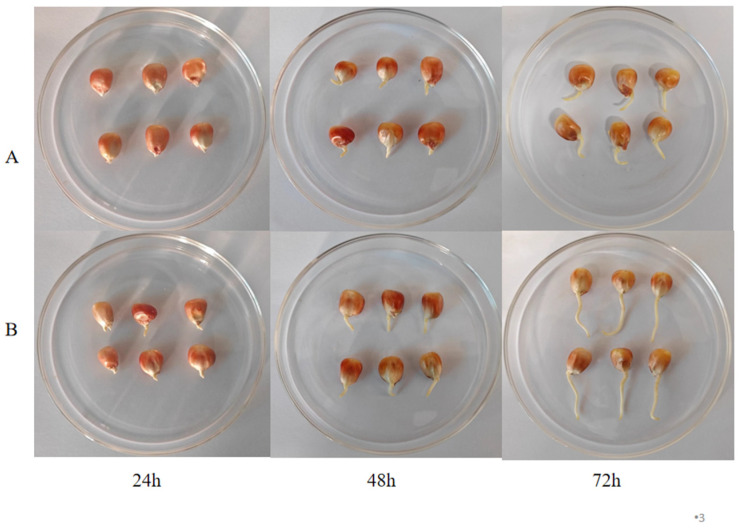
Physical changes in maize during germination. (**A**) control group; (**B**) pulsed light group.

The growth of maize in the PL treatment group was notably better than in the CK group. At 24 h, the PL-treated maize had already sprouted, whereas maize in the CK group remained at the swelling stage (Figure 1), indicating a positive effect of PL treatment on maize germination. Exposure to intense light stress causes seeds to accumulate excessive reactive oxygen species, leading to increased membrane lipid peroxidation and delipidation. This damage to the cell membrane structure significantly reduces the membrane’s ability to resist water penetration, facilitating the movement of water molecules through the cell wall. Additionally, this condition accelerates the nutrient transfer rate, enabling the embryo to absorb water more effectively, which in turn promotes its development and growth [25].

**Figure 2:**
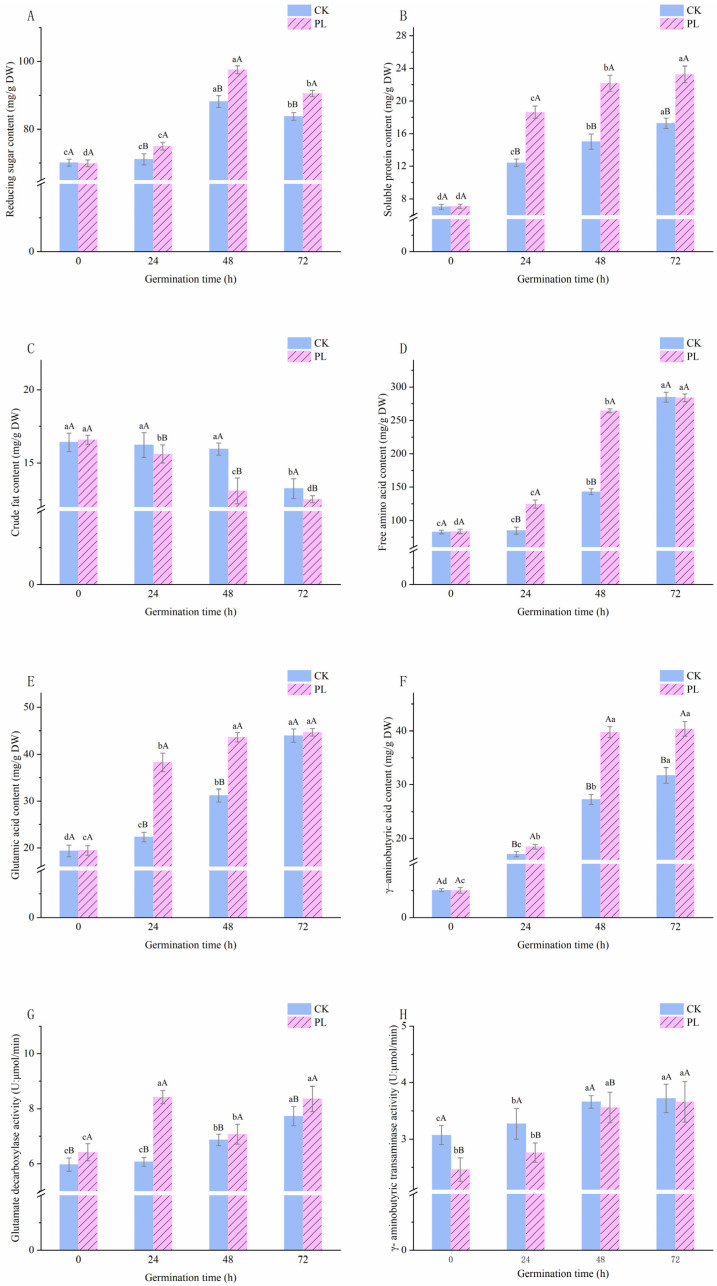
Changes in metabolite and enzyme activities during maize germination. (**A**) Reducing sugar content; (**B**) soluble protein content; (**C**) crude fat content; (**D**) free amino acid content; (**E**) glutamic acid content; (**F**) *γ*–Aminobutyric acid content; (**G**) glutamate decarboxylase activity; (**H**) *γ*–Aminobutyric transaminase activity. Different lowercase letters indicate significant differences (*p* < 0.05) in the data for the same treatment group at different germination times. Different capital letters indicate significant differences (*p* < 0.05) between the pulsed light group and control group at the same germination time. PL: pulsed light; CK: control.

Both the PL-treated and CK groups exhibited similar patterns of increasing and then decreasing sugar content during germination. The peak reducing sugar content in maize occurred at 48 h of germination, reaching 97.54 ± 1.14 mg/100 g in the PL group and 88.14 ± 1.74 mg/100 g in the CK group, respectively. However, after 72 h of treatment, the PL group demonstrated an 8.11% increase in reducing sugar content compared to the CK group (90.56 ±1.14 mg/100 g vs. 83.77 ±0.96 mg/100 g) (Figure 2A). Soluble protein content in the PL group (23.26 ± 1.03 mg/100 g) was 34.76% higher than in the CK group (17.26 ± 0.64 mg/100 g) 72 h after germination; however, this difference was not significant (Figure 2B). The trend in crude fat content was similar for both groups, showing a decrease as germination progressed (Figure 2C). The crude fat content in the PL group decreased more rapidly, accelerating after 24 h and reaching 75.59% of its initial content by 72 h. By contrast, the CK group showed no significant change in fat content during the first 24 h (Figure 2C).

During maize germination, significant changes in soluble sugar content across both groups suggest active synthesis and metabolism driven by enzymes converting starch into sugars, with more pronounced effects in the PL group indicating enhanced carbohydrate metabolism after 24 h. Sugar content was consistently higher and fatty acid content was consistently lower in PL-treated plants compared to control plants (Figure 2A,C). Considering the faster rate of germination in PL-treated seeds, it can be inferred that fatty acid catabolism is initiated early, between 48 and 72 h, to increase carbohydrate availability for the germinating embryo—the main source of energy for most cereal seeds during germination [26,27]. PL treatment also increased soluble protein levels by breaking down storage proteins and synthesizing new ones, facilitating rapid germination. Collectively, these data suggest that PL treatment not only promotes but accelerates germination, with the PL group consuming more energy—proteins, sugars, and fats—possibly in response to stress.

### 3.2. Effect of Pulsed Light Treatment on Free Amino Acids and Glutamic Acid Content in Germinated Maize

FAAs content in both the CK and PL groups steadily increased throughout germination (Figure 2D). For example, between 24 h and 72 h, FAAs content in the PL group was significantly higher than that of the CK group (*p* < 0.05), rising from 83.55 ± 3.26 mg/100 g to 283.78 ± 5.78 mg/100 g. After 72 h, the rate of increase in the PL group plateaued while it continued to rise in the CK group. Glu content mirrored the trend of FAAs content, with the rate of increase in the PL group beginning to slow after 48 h (Figure 2E). Between 24 h and 72 h, the Glu content in the PL group was significantly higher than in the CK group, peaking at 44.67 ± 0.78 mg/100 g at 72 h, representing an 11.79% increase compared to the CK group (39.96 ± 1.42 mg/100 g).

Increases in FAAs content in the PL group have also been observed under other exogenous physical conditions, such as UV light [28], hydrostatic pressure [29], and ultrasonic treatments [30]. These studies consistently demonstrate enhancements in seed germination and the enrichment of functional factors. Glu, a key substrate for GABA synthesis in plant cells, significantly influences GABA production [22]. Free Glu is derived from three main pathways: protein hydrolysis, the Glu synthetase cycle, and the Glu dehydrogenase cycle [26]. Similar to fatty acid breakdown, the increase in Glu content (Figure 2E) indicates that maize initiates germination and growth more rapidly after PL treatment, which is accompanied by a relatively earlier acceleration in catabolic activities that free up Glu.

### 3.3. Effect of Pulsed Light Treatment on Glutamic Acid Decarboxylase Activity, γ–Aminobutyric Transaminase, and γ–Aminobutyric Acid Content in Germinated Maize

The GAD activities of germinated maize in both groups exhibited an “increasing–decreasing–increasing” trend, with a peak value of 8.42 ± 0.24 U in the PL group at 24 h of germination and 7.02 ± 0.21 U in the CK group at 48 h, significantly higher than in the CK group (*p* < 0.05) throughout germination (Figure 2F). The GABA–T activity of germinated maize in both groups showed a gradual increase, peaking at 72 h with levels of 3.16 ± 0.36 U in the PL group and 3.72 ± 0.25 U in the CK group (Figure 2G). The GABA–T activity of the PL group was significantly lower than that of the CK group throughout germination (Figure 2G). The GABA content increased continuously during germination, with the PL group consistently recording higher levels than the CK group (Figure 2H). At 48 h, the GABA content peaked at 39.77 ± 1.01 mg/100 g in the PL group. At 72 h, the GABA content peaked at 40.36 ± 1.33 mg/100 g in the PL group, which was 8.01 times higher than in ungerminated maize and 27.20% higher than in germinated maize in the CK group (Figure 2H).

As the substrate for GABA synthesis in plant cells, Glu concentrations directly influence GABA production. GABA, an essential intermediate in the branching pathway of the tricarboxylic acid cycle, is produced from Glu through catalytic decarboxylation by GAD in plants. In this pathway, GABA is transaminated to succinate semialdehyde by GABA–T, which is further converted to succinate to enter the tricarboxylic acid cycle. Together, the activities of GAD and GABA–T regulate *γ*–aminobutyric acid metabolism [31]. In this study, GAD activity in both groups coincided with the most rapid increases in Glu content, whereas decreased GAD activity was correlated with slower increases in Glu content. Glu not only serves as a precursor for GABA synthesis but also influences GAD activity [32,33]. GAD is a Ca^2+^/calmodulin-dependent enzyme with a calmodulin-binding region [34]. PL treatment was shown to promote the degradation of substances around the plant cell wall and increase the intracellular Ca^2+^ concentration, contributing significantly to the enhanced GAD activity in the PL group. Additionally, PL treatment was found to cause intracellular damage by reducing cytoplasmic pH and inhibiting GABA–T activity [35]. In summary, PL treatment may enhance GABA enrichment in plants by promoting the degradation of substances surrounding the plant cell wall, increasing intracellular Ca^2+^ concentration, and boosting GAD activity.

### 3.4. Metabolomics Analysis of Pulsed Light Treatment on γ–Aminobutyric Acid Enrichment in Germinated Maize

#### 3.4.1. Liquid Chromatography Mass Spectrometry/Mass Spectrometry analysis

**Figure 3:**
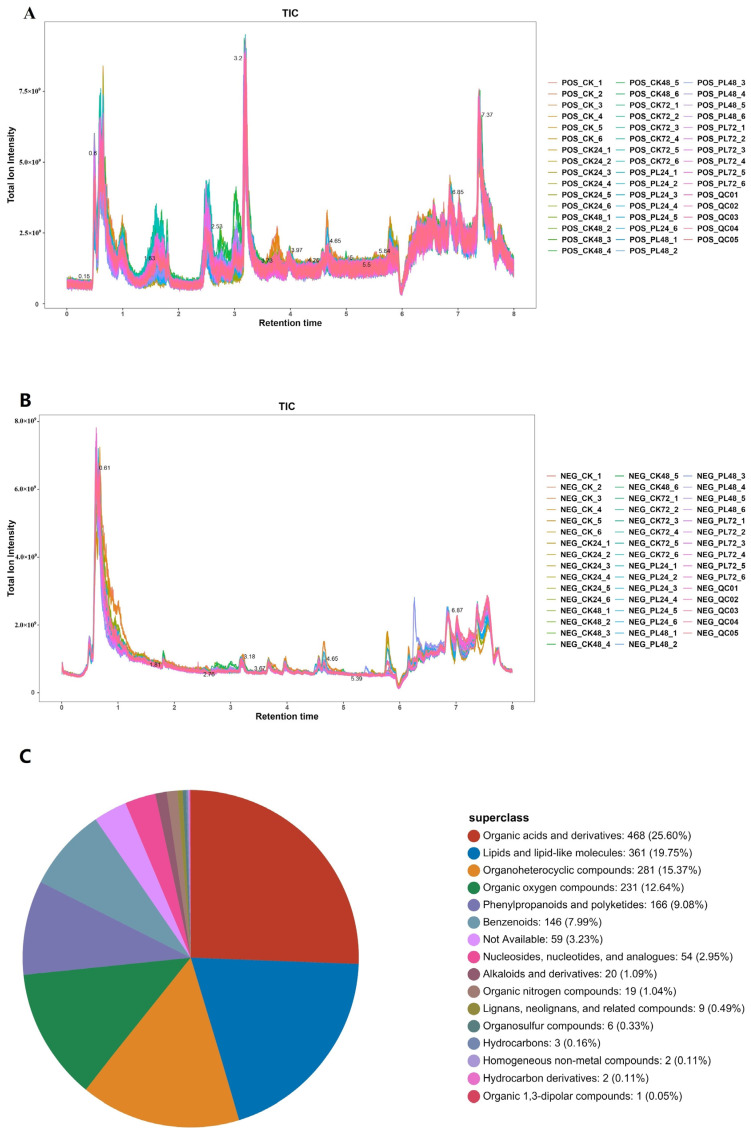
Metabolite Profiling of Germinating Maize. (**A**) Total ion flow diagram in positive ion mode. (**B**) Total ion flow diagram in negative ion mode. (**C**) Proportion of metabolite classes. PL: pulsed light; CK: control.

A non-targeted metabolomics technique based on LC–MS/MS was used to detect the metabolites in germinated maize, and the total ion flow diagram in positive and negative ion modes is shown in Figure 3A and Figure 3B, respectively. A total of 2130 metabolites were identified—1237 in positive ion mode and 893 in negative ion mode. These metabolites were classified into 16 categories (Figure 3C). The major categories included organic acids and derivatives (26.60%), lipids and lipid-like molecules (19.75%), organoheterocyclic compounds (15.37%), organic oxygen compounds (12.64%), and phenylpropanoids and polyketides (9.08%).

While the types of compounds in all groups of germinated maize samples were similar, their levels varied significantly. The metabolites measured in both positive and negative ion modes showed differences. Organic acids, derivatives, lipids, and lipid-like molecules—primarily originating from the maize embryo—play crucial roles in protein and lipid metabolism. Additionally, organoheterocyclic compounds, organic oxygen compounds, phenylpropanoids, and polyketides serve as intermediates in carbohydrate and amino acid metabolism, as well as the biosynthesis of various secondary metabolites. These intermediates are primarily derived from the mobilization of starch storage and the degradation of certain proteins in the endosperm during seed germination. Han et al. [36] observed similar dynamics in metabolite profiles during wheat germination, confirming the consistency of these biochemical processes across different cereals.

#### 3.4.2. Principal Components Analysis and Partial Least Squares Discriminant Analysis of the Metabolites

PCA analysis of the metabolite profiles of maize seeds with different germination treatments revealed that all treatment groups could be clustered into a single group. Notably, PL24 was closer to CK24 and clearly separated from the other groups, indicating that the differences in pre-germination maize metabolites were not significant, consistent with previous reports [37].

**Figure 4:**
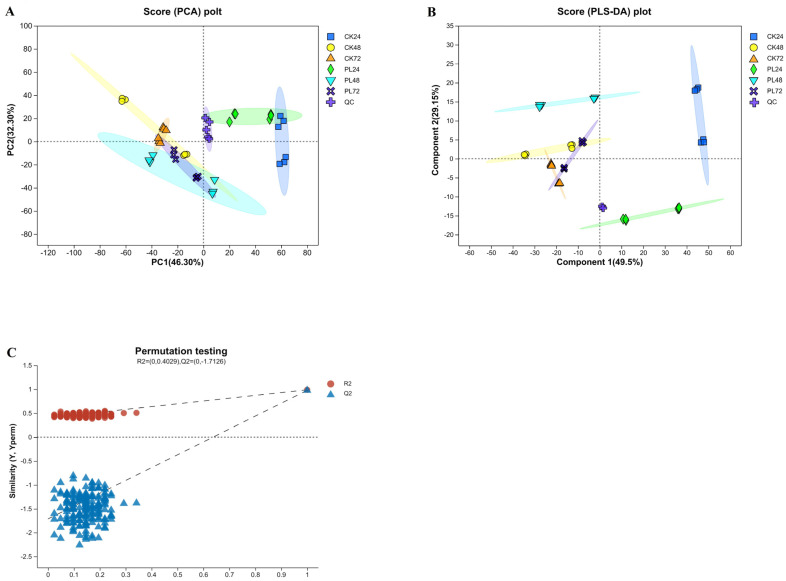
Multivariate analysis of metabolites in germinated maize. (**A**) Score chart of principal component analysis in combined positive and negative ion modes. (**B**) Partial least squares discriminant analysis in combined positive and negative ion mode. (**C**) Permutation testing of the partial least squares discriminant analysis model in combined positive and negative ion mode. PL: pulsed light; CK: control; QC: quality control.

However, the two groups of samples were in different quadrants, indicating a significant effect of PL treatment on maize germination metabolism. Further analysis with PLS–DA showed that CK24 and PL24 were significantly separated from the other groups (Figure 4B), suggesting that later stages of maize germination exhibited more metabolic variations than earlier stages. PL48 exhibited a pronounced outlier trend compared to CK48, PL72, and CK72, positioning it in a different quadrant from CK24 and PL24. This indicates that with PL treatment, the most significant metabolic changes occurred 48 h after germination. The PCA and PLS–DA analysis plots showed a dense distribution of QC samples, suggesting a high degree of experimental reproducibility. Samples within groups were more aggregated with less variability, all falling within the 95% confidence interval, suggesting minimal differences between replicates and validating the sampling method. For the PLS–DA model, the parameters for the positive and negative ion models were R2 = 0.4029 and Q2 = −1.7126, respectively (Figure 4C). The R2 exceeding the Q2 demonstrates the method’s ability to distinguish between the PL and CK groups at various germination times without overfitting, enabling further analysis of differential metabolites in germinated maize.

#### 3.4.3. Analysis of Differential Metabolites in Germinated Maize

**Figure 5:**
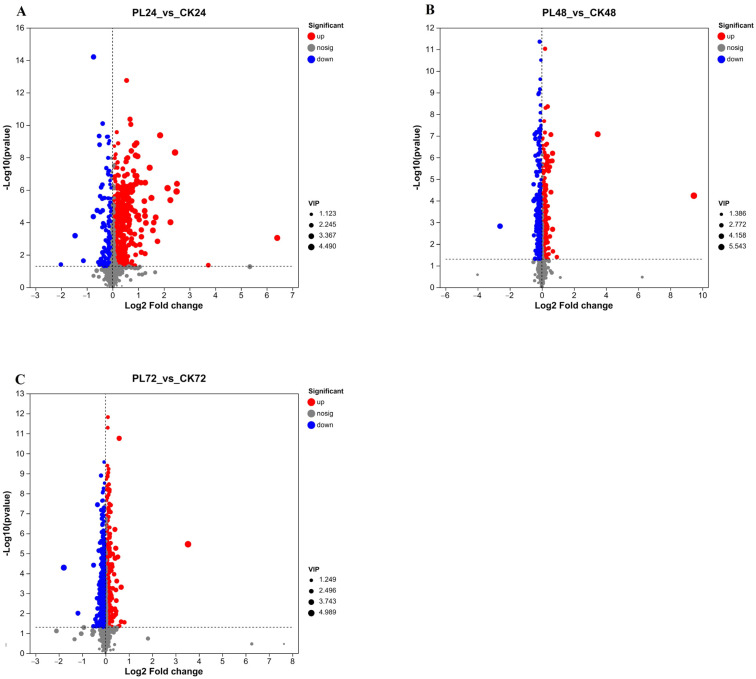
Volcanic map of different metabolites in germinated maize under different treatments. The data are displayed in combined positive and negative ion mode. The x-axis shows the log2 fold change, representing the multiple change in each metabolite, while the y-axis shows −log10 (FDR-value) from the Student’s *t*-test. Red dots indicate up-regulated metabolites, blue dots indicate down-regulated metabolites, and gray dots indicate metabolites with no significant difference. (**A**) PL24 vs Ck24, (**B**) PL48 vs CK48, (**C**) PL72 vs CK72. PL: pulsed light, CK: control. the horizontal coordinate of the bubble is the enrichment factor, which indicates the size of the influence factor of the pathway in the topological analysis, and the size of the bubble indicates the number of metabolites in the pathway, and the larger the bubble indicates the greater number of differential metabolites in the pathway.

**Figure 6:**
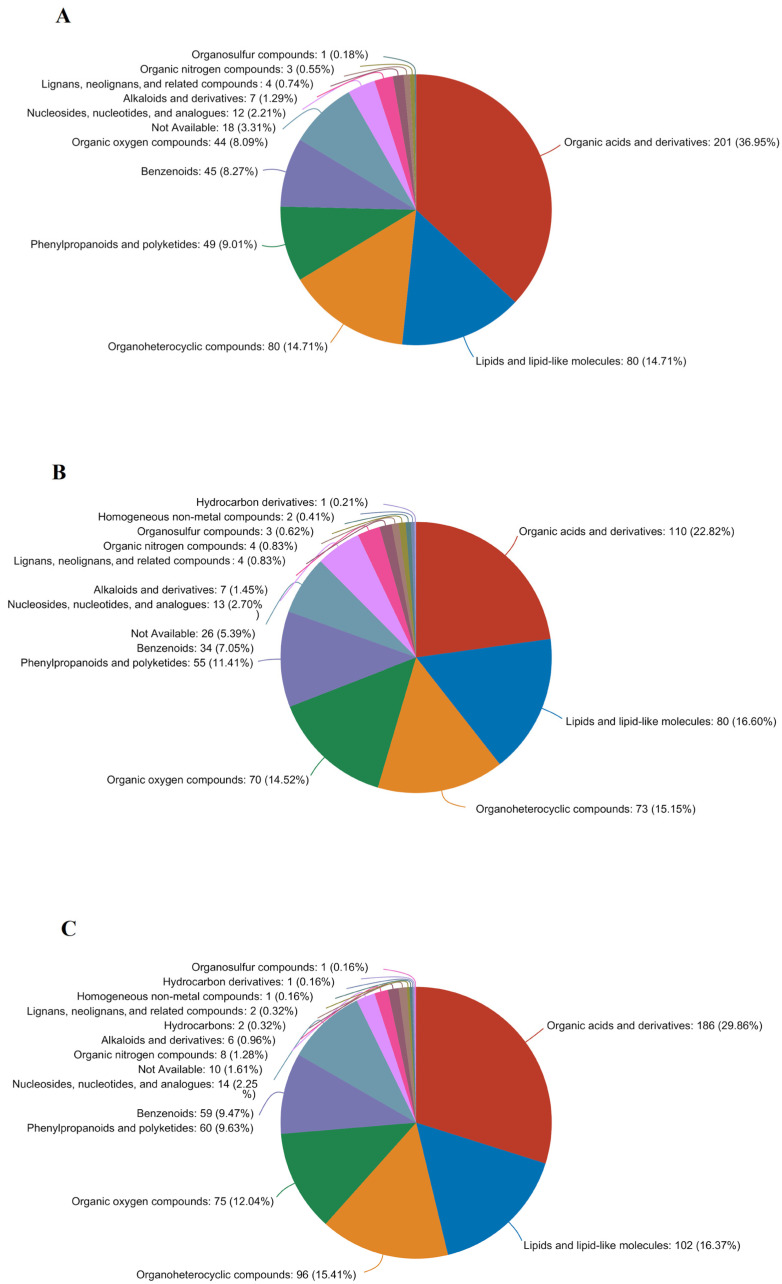
Pie chart of differential metabolite classification. (**A**) PL24 vs. CK24; (**B**) PL48 vs. CK48; (**C**) PL72 vs. CK72. PL: pulsed light; CK: control.

To delve deeper into the impact of PL treatments on maize metabolites during germination, VIP values were calculated. The analysis integrated the PLS–DA model and one-way ANOVA to identify differential metabolites, focusing on those with fold changes ≥ 2 and ≤0.5, VIP scores > 1.0, and significance (*p* < 0.05 in the Student’s *t*-test). In the PL24 vs. CK24 comparison, a total of 568 significant differential metabolites were identified, with 689 up-regulated and 121 down-regulated (Figure 5A). These significant differential metabolites were categorized into 12 groups: organic acids and derivatives (201 metabolites, 36.95%), lipids and lipid-like molecules (80 metabolites, 14.71%), organoheterocyclic compounds (80 metabolites, 14.71%), phenylpropanoids and polyketides (49 metabolites, 9.01%), benzenoids (45 metabolites, 8.27%), organic oxygen compounds (44 metabolites, 8.09%), unspecified substances (18 metabolites, 3.31%), along with sulfur nitrogen compounds, lignin, alkaloids, nucleic acids, and their analogs (Figure 6A).

In the PL48 vs. CK48 comparison, 300 differential metabolites were identified, with 114 up-regulated and 186 down-regulated (Figure 5B). These metabolites were classified into 13 categories: 110 (22.82%) organic acids and derivatives, 80 (16.60%) lipids and lipid-like molecules, 70 (14.52%) organic oxygen compounds, 55 (11.41%) phenylpropanoids and polyketides, 34 (7.05%) benzenoids, and 26 (7.05%) unspecified substances, among others, including nucleic acids and their analogues, alkaloids, lignin, sulfur–nitrogen compounds, hydrocarbons, and non-metallic compounds (Figure 6B).

In the PL72 vs. CK72 comparison, a total of 438 differential metabolites were identified, with 131 up-regulated and 307 down-regulated (Figure 5C). These metabolites were classified into 15 categories: 186 (29.86%) organic acids and derivatives, 102 (16.37%) lipids and lipid-like molecules, 96 (15.41%) organoheterocyclic compounds, 75 (12.04%) organic oxygen compounds, 60 (9.63%) phenylpropanoids and polyketides, 59 (9.47%) benzenoids, 14 (2.25%) nucleosides, nucleotides, and analogs, 10 (1.61%) unspecified substances, and others, including sulfur–nitrogen compounds, alkaloids, hydrocarbons, lignin, and non-metallic compounds (Figure 6C).

The metabolites that differed between the PL and CK groups included organic acids and derivatives, lipids, lipid-like molecules, and organoheterocyclic compounds. Energy metabolism intermediates such as GABA, 4–acetaminobutyric acid, arginine, succinic acid semialdehyde, and glutamine were up-regulated, whereas 5–pyridoxolactone, salicyluric acid, 2–isopropylmalic acid, tyrosyl–valine, and adenosine were down-regulated (Figure 6). These metabolites are crucial for plant adaptation to various stresses and are involved in key biosynthesis pathways, including hydrogenation, decarboxylation, and substitution [38]. Lipids and lipid-like molecules, serving as essential energy sources, are concentrated in the pasty and germ layers of cereals. They provide calories for grain germination and regulate grain metabolism [36]. Organoheterocyclic compounds, important secondary metabolites in plants, contribute to growth, development, and stress responses [39]. Our findings indicate that there were more differential metabolites in the middle and late stages of maize germination compared to the pre-germination stage, reflecting the increased metabolic activities during these stages. PL treatment significantly altered maize metabolism in the pre-germination stage, leading to a greater number of differential metabolites compared to the mid-germination stage.

#### 3.4.4. Kyoto Encyclopedia of Genes and Genomes Metabolic Pathway Analysis

**Figure 7:**
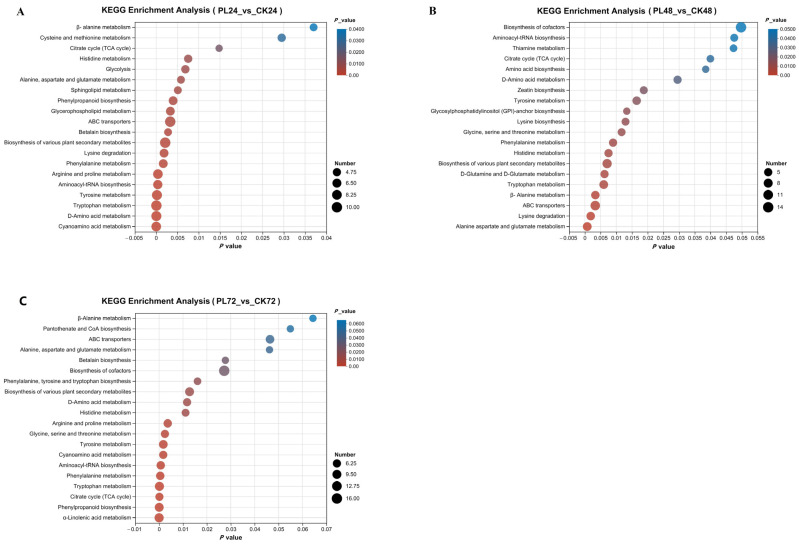
Bubble map of KEGG metabolic pathway enrichment. The horizontal coordinate represents the enrichment significance (*p*-value), and the vertical coordinate represents the KEGG pathway. The size of the bubbles indicates the number of metabolites enriched in each pathway. The size of each bubble indicates the number of metabolites enriched in each pathway. (**A**) PL24 vs. Ck24, (**B**) PL48 vs. CK48, (**C**) PL72 vs. CK72. PL: pulsed light, CK: control.

The KEGG Pathway database was used to analyze the involvement of differential metabolites in metabolic pathways. The top 20 pathways, sorted by *p*-value, were selected to create an enrichment bubble diagram (Figure 7). In the PL24 vs. CK24 comparison, 43 metabolites were up-regulated, 13 were down-regulated, and 11 were unchanged (Figure 7A). Seven pathways were associated with GABA anabolism: alanine, aspartate, and glutamic acid metabolism; D–amino acid metabolism; histidine metabolism; *β*–alanine metabolism; galactose metabolism; the tricarboxylic acid cycle (TCA cycle); and lysine biosynthesis. PL treatment significantly up-regulated key metabolites, including GABA, L–glutamic acid, *α*–ketoglutaric acid, histidine, and lactose, whereas citrate, oxalosuccinate, galactitol, and Glu were significantly down-regulated, with Glu showing the most significant increase.

In the PL48 vs. CK48 comparison, 42 metabolites were up-regulated, 16 were down-regulated, and 13 were unchanged (Figure 7B). Nine metabolic pathways were associated with GABA synthesis, including amino acid biosynthesis, including D–glutamine and D–glutamate metabolism, and glycine, serine, and threonine metabolism. PL treatment significantly up-regulated GABA, L–glutamic acid, *α*–ketoglutaric acid, lysine, and mesaconic acid, whereas citrate, glutamine, serine, glutamate, succinic acid semialdehyde, and glutamic acid semialdehyde were down-regulated. GABA content increased most significantly.

In the PL72 vs. CK72 comparison, 44 metabolites were up-regulated, 19 were down-regulated, and 13 were unchanged (Figure 7C). Six metabolic pathways were associated with GABA synthesis: *β*–alanine metabolism, alanine, aspartate, and glutamic acid metabolism, D–amino acid metabolism, histidine metabolism, glycine, serine, and threonine metabolism, and the TCA cycle. PL treatment significantly up-regulated GABA, L–glutamic acid, 1–glucose phosphate, and arginine, whereas Glu, L–valine, ornithine, argininosuccinic acid, and histidine were down-regulated.

**Figure 8:**
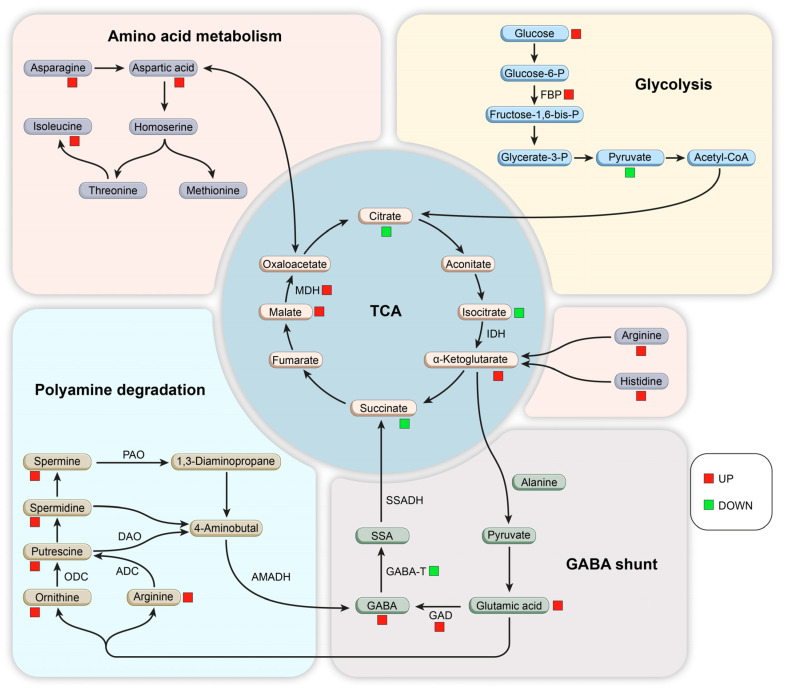
Model for the effect of PL treatment on *γ*–aminobutyric acid metabolism in germinating maize. Red squares indicate significant up-regulation of metabolite content, and blue squares indicate significant down-regulation of metabolite content. FBP: fructose 1,6–bisphosphatase; MDH: malate dehydrogenase; IDH: isocitrate dehydrogenase; TCA: tricarboxylic acid cycle; SSADH: succinic semialdehyde dehydrogenase; GABA–T: *γ*–aminobutyric transaminase; GAD: glutamate decarboxylase; GABA: *γ*–aminobutyric acid; SSA: succinic semialdehyde; AMADH: aminoaldehyde dehydrogenase; PAO: polyamine oxidase; DAO: diamine oxidase; ODC: ornithine decarboxylase; ADC: arginine decarboxylase.

Based on the above results, a graph was drawn to show the effect of PL treatment on the GABA synthesis and metabolism pathways in germinated maize (Figure 8), providing a possible explanation for the mechanism of GABA enrichment. PL treatment significantly influenced amino acid metabolism during maize germination, impacting pathways such as alanine, aspartate, and glutamic acid metabolism, as well as the synthesis and metabolism of D–amino acids, histidine, *β*–alanine, lysine, D–glutamine, and D–glutamate, along with glycine, serine, and threonine. Notably, after PL treatment, maize exhibited a significant increase in Glu content and a decrease in glutamate levels, enhancing glutamine metabolism. The treatment also improved the permeability of maize cell membranes, boosting Glu interaction with GAD, thereby increasing GABA production. Additionally, amino acids like ornithine, arginine, and histidine, integral to the TCA cycle [40], transform through enzymatic actions affecting glutamic acid metabolism [41]. Specifically, arginine’s conversion to ornithine and subsequent decarboxylation to putrescine lead to GABA production, enhancing its accumulation in maize [42]. Furthermore, arginine and histidine conversions produce *α*–ketoglutarate, which not only participates in the TCA cycle but also reacts with alanine to form pyruvate, influencing overall glutamic acid metabolism [43]. Carbohydrate metabolic pathways, including the TCA cycle, galactose metabolic pathway, and carbon metabolic pathway, play vital roles in GABA synthesis. Succinic acid, a product of the TCA cycle, is downstream of *γ*–aminobutyric acid metabolism. Citrate, an end product of the TCA cycle, was significantly downregulated by PL treatment, indicating an inhibition of GABA conversion to citrate, favoring GABA accumulation. In the glycolytic pathway, glucose breaks down into pyruvate, entering the mitochondria for oxidation and decarboxylation to produce acetyl coenzyme A, a key intermediate for the conversion of sugars, fats, and proteins in the organism [37]. The dual effect of PL on maize amino acid and carbohydrate metabolism led to a significant up-regulation of GABA during mid-germination, consistent with earlier findings [44]. Additionally, PL treatment altered plant hormone signal transduction, energy metabolism, pyrimidine metabolism, and ABC transporters in germinated maize. These pathways indirectly contribute to GABA enrichment by influencing the involved metabolites. For instance, pyrimidine metabolism is linked to aspartic acid, which regulates the synthesis rate of pyrimidine nucleotides via aspartic acid aminomethyltransferase [45]. Transporter proteins are essential for mediating the transport of various molecules, playing a critical role in plant growth and development [46]. While these pathways are not directly involved in GABA synthesis, they can indirectly contribute to its enrichment in germinated maize.

## 4. Conclusions

This study investigated the impact of PL treatment on the chemical composition and metabolomics of germinated maize, employing quantitative analysis and non-targeted metabolomics. The experimental results showed that the content of GABA in germinated maize could be increased by 27.20% after PL treatment. A total of 2130 metabolites were identified through non-targeted metabolomics analysis: GABA, 4–acetaminobutyric acid, arginine, succinic acid semialdehyde, and glutamine were up-regulated, whereas 5–pyridoxolactone, salicyluric acid, 2–isopropylmalic acid, tyrosyl–valine, and adenosine were down-regulated. PL treatment has significant effects on amino acid metabolism, carbohydrate metabolism, plant hormone signal transduction, energy metabolism, pyrimidine metabolism, and ABC transporters in maize. This study illuminates how PL treatment influences maize seed metabolism, identifying specific metabolites and pathways crucial for GABA enrichment. These results strongly support the proposed mechanism by which PL treatment enhances maize germination and GABA enrichment. The research results indicate that PL treatment accelerates maize nutrient breakdown and modulates key metabolic processes essential for GABA synthesis. This research expands the potential applications of PL technology in promoting health-enhancing maize products, providing a technical reference and theoretical basis for developing functional maize-based health foods.

## Data Availability

The original contributions presented in the study are included in the article, further inquiries can be directed to the corresponding author.
